# Serum neurofilament light chain in myasthenia gravis subgroups: An exploratory cohort and case–Control study

**DOI:** 10.3389/fneur.2022.1056322

**Published:** 2023-01-11

**Authors:** Frauke Stascheit, Annette Aigner, Philipp Mergenthaler, Benjamin Hotter, Sarah Hoffmann, Sophie Lehnerer, Christian Meisel, Andreas Meisel

**Affiliations:** ^1^Department of Neurology, Charité—Universitätsmedizin Berlin, Corporate Member of Freie Universität Berlin and Humboldt-Universität zu Berlin, Berlin, Germany; ^2^NeuroCure Clinical Research Center, Charité—Universitätsmedizin Berlin, Corporate Member of Freie Universität Berlin, Humboldt-Universität zu Berlin, Berlin, Germany; ^3^Institute of Biometry and Clinical Epidemiology, Charité—Universitätsmedizin Berlin, Corporate Member of Freie Universität Berlin, Humboldt-Universität zu Berlin, Berlin, Germany; ^4^Center for Stroke Research Berlin, Charité—Universitätsmedizin Berlin, Corporate Member of Freie Universität Berlin, Humboldt-Universität zu Berlin, Berlin, Germany; ^5^Berlin Institute of Health (BIH), Berlin, Germany; ^6^Department of Immunology, Institute of Medical Immunology, Charité—Universitätsmedizin Berlin, Berlin, Germany; ^7^Labor Berlin, Charité Vivantes GmbH, Berlin, Germany; ^8^Integrated Myasthenia Gravis Center, Charité—Universitätsmedizin Berlin, Corporate Member of Freie Universität Berlin, Humboldt-Universität zu Berlin, and Berlin Institute of Health, Berlin, Germany

**Keywords:** serum neurofilament light chain, myasthenia gravis, antibody status, biomarker, disease severity

## Abstract

**Background:**

This study aimed to evaluate the association of neurofilament light chain (Nfl) with neuromuscular destruction and disease severity in the serum of patients with myasthenia gravis (MG).

**Materials and methods:**

Sera from 134 patients with MG with varying degrees of disease severity and autoantibody (Abs) status were analyzed and compared to controls in a cross-sectional design. Prospectively, we additionally measured serum NfL (sNfl) levels in patients with MG longitudinally for up to 3 years. Based on linear regression, differences between patients and controls were assessed. With correlation coefficients and mixed linear regression, the association among sNfl levels, socio-demographics, disease activity (Quantitative Myasthenia Gravis (QMG) score and Myasthenia Gravis Activities of Daily Living (MG-ADL) scale), Abs-status (acetylcholine receptor antibody (AChR-Abs), muscle-specific receptor tyrosine kinase antibody (MuSK-Abs), lipoprotein-related protein 4 (LRP4), and seronegative), Abs titer, treatment regime (pyridostigmine, steroids, and immunosuppressive therapies), and thymectomy were investigated.

**Results:**

sNfl levels were higher in patients with MG compared to controls (median: 11.2 vs. 7.88), where sNfl levels were highest in anti-AChR-Abs positive patients (median 12.6), followed by anti-MuSK-Abs positive, anti-LRP4-Abs positive, and seronegative patients. Adjusting for age and sex, sNfl levels of patients with MG were on average 35% higher compared to controls (35.1, 95% CI: 8.4;68.3) and highest for patients with seronegative MG (44.35; 95% CI 16.47; 78.90). We found no relevant relationship between individual changes in sNfl and changes in QMG and MG-ADL scores.

**Conclusion:**

sNfl levels are higher in patients with MG than in controls but were not consistently associated with clinical severity. Thus, sNfl is not a suitable biomarker to monitor individual disease progression in patients with MG.

## 1. Introduction

Myasthenia gravis (MG) is an autoimmune disease affecting the neuromuscular junction (NMJ) by specific autoantibodies (1, 2). While the effector mechanisms of the disease disturbing the functions of the NMJ are relatively well known, the etiology of MG and its heterogeneity in the clinical course are poorly understood. Importantly, there is an urgent need for sensitive biomarkers in MG predicting treatment responses and outcomes, especially in light of emerging and more specific treatment options (3, 4).

Neurofilaments (Nfl) are important structural elements of neurons and are released into the extra-cellular environment upon neuronal injury (5). Nfl has been studied in several neurodegenerative and central neuroinflammatory conditions (6–12).

Less is known about the role of Nfl in peripheral nervous system disorders, but there is increasing evidence that sNfl potentially has diagnostic and prognostic value in acquired polyneuropathies (13, 14), inherited peripheral neuropathy (15), and Guillain-Barré syndrome (16).

Although MG is not a typical disorder characterized by neuronal injury, histopathological studies in patients with MG demonstrate neurogenic changes regardless of MG subtype (17, 18). Additionally, it is known that antibodies (Abs) against the acetylcholine receptor (AChR), the muscle-specific kinase (MuSK), and the lipoprotein-related protein 4 (LRP4) are directly pathogenic, inducing accelerated degradation of these receptors (19–21) and leading to local membrane damage at the NMJ (22). These mechanisms destabilize the signaling pathways at the NMJ, leading to ACh deprivation, which is crucial for proper muscle innervation (23).

As the neuromuscular terminal seems to be enriched with proteins critical for NMJ structure and function, including cytoskeleton-associated proteins like Nfl (24–27), the different extent of neuromuscular destruction on the NMJ could have a measurable effect on Nfl release (28, 29). Here, we investigate whether sNfl is increased in patients with MG compared to controls and whether sNfl could serve as a biomarker of disease severity.

## 2. Materials and methods

### 2.1. Standard protocol approvals, registration, and patient consent

The study was approved by the ethics committee of the Charité—Universitätsmedizin Berlin (EA1/281/10). All patients gave written informed consent in accordance with the Declaration of Helsinki in its currently applicable form. The study followed the Strengthening the Reporting of Observational Studies in Epidemiology (STROBE) reporting guidelines (Supplementary material).

### 2.2. Study design

This is an explorative cohort study examining sNfl levels in patients with MG with different Abs statuses. Longitudinally we followed the patients and assessed sNfl as a potential biomarker for MG disease severity as measured by the Quantitative Myasthenia Gravis (QMG) score and Myasthenia Gravis Activities of Daily Living (MG-ADL) scale. Based on the cohort design, patients were matched with controls at baseline.

### 2.3. Patients and controls

This study was carried out at the certified integrated Center for Myasthenia gravis (iMZ) of the Charité—Universitätsmedizin Berlin, Germany. Patients over the age of 18 years with a confirmed diagnosis of MG, based on the current guidelines of the German Neurological Society (30), were included independent of disease duration and severity. Patients with MG were consecutively screened at the iMZ clinic from March 2011 to October 2020. Due to the potential influence on sNfl levels, patients with a previous history of cancer were excluded, excepting thymoma (31). Other exclusion criteria were diagnosis of polyneuropathy, neurodegenerative diseases (atypical and typical parkinsonian disorders, frontotemporal dementia, Alzheimer's disease, and amyotrophic lateral sclerosis (ALS)), central nervous inflammatory diseases (multiple sclerosis and autoimmune encephalitis), traumatic brain injury, and previous stay on intensive care unit (< 3 months) (32). Overall, 134 patients could be included for baseline analysis and were followed up to 3 years. For exploratory longitudinal analysis, we tested sNfl in a total of 59 patients. Age- and sex-matched controls without comorbidities recruited from the clinical staff of the Charité-Universitätsmedizin Berlin were enrolled as a control group (*n* = 31).

### 2.4. Clinical assessment

Patients were categorized into subgroups according to their Abs status (anti-AChR-Abs, anti-MuSK-Abs, anti-LRP4-Abs, and seronegative). None of the patients were double positive for an MG-Abs. Age, sex, disease duration, history of myasthenic crisis, current MG-specific medication (cholinesterase inhibitors, glucocorticoids, and long-term immunosuppressants), history of thymectomy, and comorbidities were collected. Disease severity was assessed using the Myasthenia gravis foundation of America (MGFA) classification, the QMG, and the MG-ADL score (33, 34). According to the MGFA classification, patients were grouped into ocular (MGFA I) or generalized patients with MG (MGFA II-IV) at the time of study inclusion and blood sampling. The MGFA classification system served as an assessment of patients' disease severity at the time of data sampling as a simple scoring system (33). The QMG score was developed as a tool for assessing disease severity as well as the pattern of deficits based on quantitative testing of sentinel muscle groups (33, 35). It is a 13-item score with a total score range of 0–39 points and shows good interrater variability (36). Its reliability and validity have been demonstrated in several studies (35, 37). The MG-ADL is an eight-question survey of symptom severity, with each response graded from 0 (normal) to 3 (most severe) (34). Questions include ocular, oropharyngeal, respiratory, and extremity functions. The total MG-ADL score ranges from 0 to 24. All scores were assessed at the time of sample collection.

### 2.5. sNfl measurement

Serum samples were collected from patients with MG and controls, clotted for 30 min at room temperature and then centrifuged, aliquoted at room temperature, and stored at −80°C. The temperature of the freezers was continuously monitored, and samples were thawed only before analysis. sNfl concentrations were measured using the SIMOA Nf-light kit^®^ in SR-S immunoassay analyzer, SIMOA™ (Quanterix Corp, Boston, MA, USA), according to the manufacturer's protocol (38). SIMOA Nf-light kit^®^ is an ultrasensitive paramagnetic bead-based enzyme-linked immunosorbent assay and is at least 125 times more sensitive than conventional immunoassays and maintains a high analytical performance (39). All samples were analyzed in one measurement in March 2021. Calibration curves for assay calibration were set up according to the manufacturer's instructions. As recommended by the manufacturer, the use of stored calibration curves for a maximum of 3 weeks has been validated in a curve storage study over 4 weeks during assay validation. Calibration curve validity was assessed during each analytical run by assaying controls of known concentrations at two different levels (low and high controls provided by the manufacturer with sNF-L concentrations ranging between 2 and 5 pg/ml and between 100 and 200 pg/ml, respectively). Assay variation over time was low as indicated by the coefficient of variation (CV) values of < 7.9 and 12% for high and low controls, respectively, during their shelf life of up to 6 months over different control lots.

### 2.6. Statistical analysis

Continuous data are presented as the median and interquartile range (IQR), and categorical variables as absolute frequencies and percentages. Age-adjusted z-scores were calculated for cases and controls based on a reference database of 4,532 healthy controls from the serum neurofilament light chain reference app (40–42). Comparisons of sNfl levels between MG subgroups and recruited controls were based on log-linear regression analysis, adjusted for age and sex to control for confounding by these variables. These models apply the natural logarithm to the dependent variable, for example, if the dependent variable is skewed. Based on them, we derive adjusted estimates of percentage change in sNfl levels comparing cases and controls additionally stratified by Abs-status. Association between sNfl levels of MG patients with clinical and laboratory assessments at baseline and during follow-up were analyzed by computing Spearman correlation and repeated measure Spearman correlation coefficients, respectively. Additionally, log-linear mixed models were used to assess the association between patient characteristics and sNfl, where again the results are derived as the percentage change in sNfl levels. All derived effect sizes are reported along with 95% confidence intervals (CI). All statistical analyses were performed using R (43) and R packages (44–48).

### 2.7. Primary research question

Are sNfl levels elevated in patients with MG compared to controls and can sNfl be used as a biomarker of neuromuscular destruction indirectly reflecting disease severity in patients with MG?

## 3. Results

### 3.1. Demographics and characteristics of patients with MG

We included 134 patients with MG and 31 controls (Table 1). The median age of patients with MG was 52.5 years (IQR 39.0–68.8), 63% (*n* = 85) were female, whereas the median age of controls was 46.0 years (IQR 39.0–58.0), and 68% were female. The median disease duration was 4.0 years (IQR 2.0–10.5). The main proportion of the study population was anti-AChR-Abs positive (*n* = 79, 59%), followed by anti-MuSK-Abs (*n* = 18, 13%) and anti-LRP4-Abs (*n* = 11, 8%), while 26 patients (19%) remained seronegative. For 37 anti-AChR-Abs positive, 14 seronegative, and 8 anti-MuSk-Abs positive patients, we had follow-up data. Median disease severity at the time of blood sampling according to the MGFA classification system was II (IQR I-II), median QMG 7 (IQR 2.75–13.0), and median MG-ADL 5 (IQR 2.0–8.0). About 25% of patients with MG (*n* = 30) had a history of myasthenic exacerbation/crisis as defined by rapid worsening of muscle weakness and potential airway compromise from ventilatory or bulbar dysfunction (49). Thymectomy as an immunomodulatory therapy has been undergone by 52 patients (39%). The majority of patients received immunosuppressive therapy at baseline either with corticosteroids monotherapy (*n* = 38, 28%) or with standard immunosuppressive therapy with azathioprine (*n* = 45; 34%), mycophenolate mofetil (*n* = 15, 11%), or methotrexate (*n* = 6, 4%). Escalation therapy with rituximab was administered to 3% (*n* = 4) of patients, of which, three of them were positive for anti-MuSK-Abs. One of the patients (anti-AChR-Abs positive) received eculizumab.

**Table 1:** Baseline characteristics and medical history of patients with myasthenia gravis and controls.

	**Total MG**	**Anti-ACHR-aBS+**	**Anti-mUsk-ABS+**	**Anti-lrp4-ABS+**	**Seronegative**	**Controls**
*n* (%)	134	79 (59%)	18 (13%)	11 (8%)	26 (19%)	3116pt
**Sex**						
Female, *n* (%)	85 (63%)	46 (55%)	15 (79%)	6 (68%)	25 (81%)	21 (68%)
Age at time point of sampling, median (IQR)	52.5 (39–68)	54 (39–70)	56 (33–63)	49 (42–58)	51 (39–60)	46.0 (39–58)
Disease duration (years), median (IQR)	4.0 (2.0–10.5)	3.0 (2.0–10.5)	6.0 (2.0–18.0)	1 (1.0–5.0)	3.5 (2.0–8.0)	–
History of myasthenic exacerbation/CRISIS, *n* (%) (missing)	32 (25%) (11)	19 (14%) (0)	9 (7%) (0)	0 (0%) (11)	4 (3%) (0)	–
MGFA classification at time point of sampling, Median (IQR) (missing)	2 (1.0–2.0) (1)	2 (1.0–2.0) (0)	2 (2.0–2.0) (0)	3 (2.0–3.0) (0)	2 (1.0–2.0) (1)	–
QMG, median (IQR) (missing)	7 (2.75–13.0) (10)	6 (3.0–12.0) (1)	7 (1.5–12.0) (0)	10 (2.5–13.75) (5)	10 (4.5–14.0) (4)	
MG-ADL-score, median (IQR) (missing)	5 (2.0–8.0) (3)	4 (1.0–7.0) (0)	5 (3.0–7.75) (0)	13 (12.25–24.5) (1)	6 (3.0–9.25) (2)	–
History of thymectomy	52 (39%)	41 (52%)	0 (0%)	3 (27%)	8 (31%)	–
Thymoma, *n* (%) (missing)	1 (2%) (76)	1 (2%) (32)	0 (0%) (18)	0 (0%) (9)	0 (0%) (17)	16pt
**MG-specific treatment at baseline**, ***n*** **(%)**						
Cholinesterase inhibitors	110 (82%)	13 (16%)	1 (6%)	3 (27%)	8 (26%)	
Corticosteroids Mono	35 (28%)	19 (14%)	7 (5%)	2 (2%)	6 (5%)	–
Azathioprine	45 (34%)	30 (38%)	3 (16.7%)	2 (18.2%)	10 (38.5%)	
Mycophenolate mofetil	15 (11%)	9 (11.4%)	3 (16.7%)	1 (9.1%)	2 (7.7%)	–
Methotrexate	6 (4%)	4 (5.1%)	1 (5.6%)	1 (9.1%)	0 (0%)	
Rituximab	4 (3%)	1 (1%)	3 (3%)	0 (0%)	0 (0%)	–
Eculizumab	1 (1%)	1 (0.7%)	0 (0%)	0 (0%)	0 (0%)	
SNfl (PG/ML), median (IQR)	11.2 (6.8–22.3)	12.6 (7.0–24.2)	9.1 (6.6–22.4)	8.7 (4.4–20.9)	12.3 (7.0–16.1)	7.8 (6.5–9.5)
Age-adjusted z-score (missing)	0.81 (–0.12, 1.62) (1)	1.18 (0.05, 1.75) (1)	0.70 (−0.49, 1.79) (0)	0.52 (−1.26, 1.36) (0)	1.04 (0.25, 1.62) (0)	0.08 (–0.66, 0.36) (0)

Results are presented as median (IQR) or *n* (%). Disease duration is the time from diagnosis until baseline.

AChR-+, acetylcholine receptor antibody positive MG-patients; IQR, interquartile range; LRP4+, lipoprotein related peptide 4 positive MG-patients; MG, myasthenia gravis; MG-ADL, MG activity of daily life score; MGFA, Myasthenia gravis foundation of America classification; MuSK+, muscle specific positive MG patients; sNfl, serum neurofilament light chain; QMG, quantitative myasthenia gravis score; –, not applicable.

### 3.2. sNfl levels in patients with myasthenia gravis compared to controls

**Figure 1:**
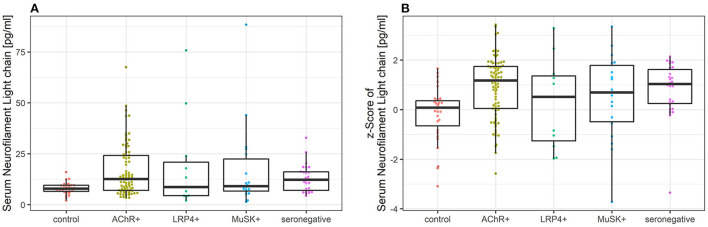
**(A)** Comparison of sNfl levels in patients with myasthenia gravis by antibody status and controls. Boxplot of sNfl levels for patients with MG by antibody status in comparison to controls. Anti-AChR-positive patients (*n* = 79) had the highest sNfl level with a median of 12.6 pg/ml (IQR 7.0–24.2), followed by seronegative (12.3 pg/ml, IQR 7.0–16.1; *n* = 26), anti-MuSK-Abs-positive patients (9.1 pg/ml, IQR 6.6–22.4; *n* = 18), and anti-LRP4-positive patients (8.7 pg/ml, IQR 4.4–20.9; *n* = 11). **(B)** Comparison of age-adjusted z-scores of sNfl levels in patients with myasthenia gravis by antibody status and controls using Boxplot of age-adjusted z-scores of sNfl levels in MG patients by antibody status in comparison to controls. The z-scores quantify the deviation of sNfl in comparison to controls of the same age based on a reference database of sNfl measured in 4,532 persons. AChR-+, acetylcholine receptor antibody positive MG-patients, Abs, antibody, IQR, interquartile range, LRP4+, lipoprotein related peptide 4 positive MG-patients, MuSK+, muscle specific positive MG patients, sNfl, serum neurofilament light chain.

**Figure 2:**
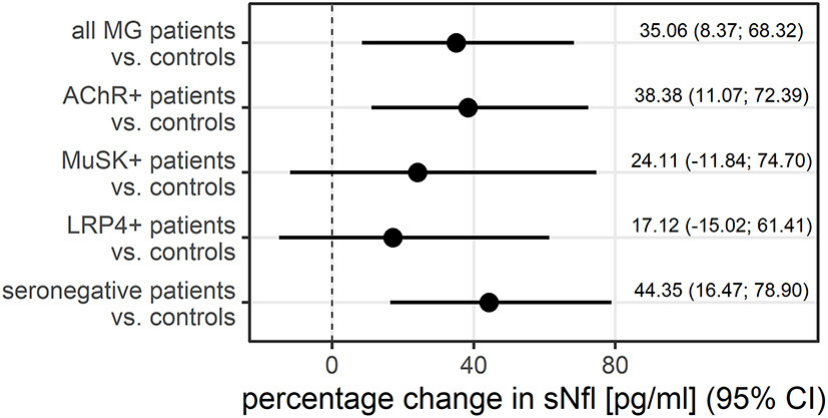
The adjusted difference between patients with MG and controls additionally stratified by antibody status, displayed as the percentage change in sNfl, derived from log-linear regression adjusted for age and sex in a logistic regression. AChR–+, acetylcholine receptor antibody positive MG-patients, CI, 95% confidence interval, LRP4+, lipoprotein related peptide 4 positive MG-patients, MuSK+, muscle specific positive MG patients, sNfl, serum neurofilament light chain.

The median sNfl levels were elevated by 3.3 pg/ml and about 1.4 times higher in patients with MG compared to controls with a median of 11.2 pg/ml (IQR 6.8–22.3) vs. 7.9 pg/ml in controls (IQR 6.5–9.5). Median sNfl levels were highest for anti-AChR-positive (median 12.6) and seronegative patients (12.3), compared to anti-MuSK-Abs (9.1) and anti-LRP4-positive patients (8.7) (Figure 1A, Table 1). Based on age-adjusted z-scores of sNfl levels, these observations were confirmed—with a median of 1.18 (IQR = 0.05, 1.75) for anti-AChR-positive, 1.04 (IQR = 0.2–1.62) for seronegative patients, 0.70 (IQR = −0.49–1.79) for anti-MuSK-Abs, and 0.52 (IQR = −1.26–1.36) for anti-LRP4-positive patients. A median of 0.08 for the recruited controls (IQR = −0.66–0.36) indicates a good similarity between the controls and the reference dataset used for the z-scores (Figure 1B, Table 1). Adjusting for age and sex, sNfl levels of patients with MG are on average 35% higher compared to controls (35.06%, 95% CI: 8.4;68.3) (Figure 2), where this difference compared to controls was even higher for seronegative patients (44.35; 95% CI 16.47; 78.90). The adjustment only for age yielded very similar estimated effects.

Additionally, we analyzed clinical characteristics of patients with MG with extensively elevated sNfl levels above 95% quantile with a median sNfl level of 49.8 pg/ml (IQR 47.5–71.6; Supplementary Table 1). Outliers were observed in all MG subgroups with higher median sNfl levels in anti-MuSK-Abs (median sNfl 66.2 pg/ml; IQR 55.2–77.3) and anti-LRP4-Abs positive patients (median sNfl of 62.75 pg/ml; IQR 56.3–69.3) compared to anti-AChR-Abs positive patients (48.6 pg/ml; IQR 47.5–58.1). In addition, disease duration was shorter, MGFA classification higher, and patients had a lower rate of thymectomy and immunosuppressive drugs. However, because sNfl is not normally distributed and the number of outliers was very low, no clear clinically relevant conclusions can be drawn from this observation.

### 3.3. sNfl levels and patient characteristics

**Figure 3:**
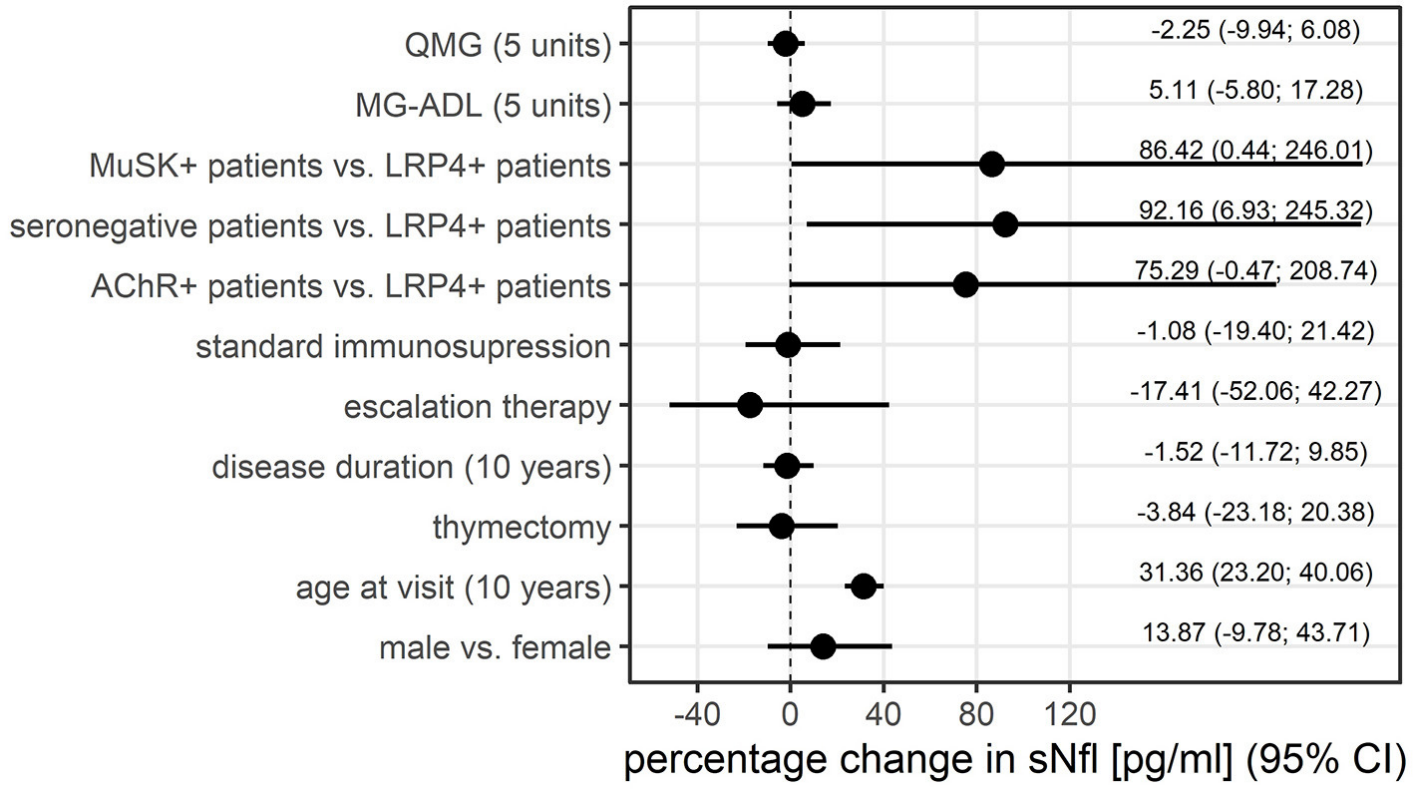
Association between patient characteristics and sNfl levels, displayed as the percentage change in sNfl, derived from mixed log-linear regression. AChR-+, acetylcholine receptor antibody positive MG-patients, LRP4+, lipoprotein related peptide 4 positive MG-patients, MG-ADL, myasthenia gravis activity of daily life score, MuSK+, muscle specific positive MG patients, sNfl, serum neurofilament light chain, QMG, quantitative myasthenia gravis score.

Older age was strongly correlated with higher sNfl levels [at baseline only: *r* = 0.68, 95% CI: 0.57; 0.79, over the entire study period: 0.43 (0.26; 0.58)], as was disease duration over the entire study period: 0.36 (0.17; 0.52), but not disease duration at baseline and MGFA score. The Spearman correlation between sNfl levels and levels of anti-AChR-Abs titer was weakly positive (0.20, −0.04; 0.43), as was for anti-MuSK-Abs levels (0.28, −0.36; 0.92) (Table 2). Based on a mixed log-linear regression model, we found that independent of other factors, male patients had 14% higher sNfl levels compared to female patients (13.9%, 95% CI: −9.8; 43.7). A 10-year increase in age is associated with ~31% (31.4, 23.2; 40.1) higher sNFl values (Figure 3).

**Table 2:** Spearman correlation coefficients for the association of patient characteristics and sNfl levels.

	**Number of observations**	**Correlation with sNfl levels spearman correlation coefficient (95% CI)16pt**
**Patient characteristics**		
Age at baseline	134	0.68 (0.57; 0.79)
Age at visit^*^	236	0.42 (0.24; 0.57)
Disease duration at baseline	134	0.01 (−0.16; 0.19)
Disease duration^*^	236	0.36 (0.17; 0.52)
Mgfa at baseline	133	0.03 (−0.16; 0.22)
Anti-AChR-ABS level	70	0.20 (−0.04; 0.43)
Anti-MuSK-ABS level	16	0.28 (−0.36; 0.92)16pt
**Disease severity**		
MG-ADL at baseline	131	0.03 (−0.14; 0.21)
MG-ADL^*^	210	−0.11 (−0.30; 0.09)
Diff MG-ADL^*^#	101	−0.20 (−0.48; 0.12)
QMG at baseline	124	0.18 (0.00; 0.36)
QMG^*^	210	−0.04 (−0.23; 0.16)
Diff QMG^*^#	77	−0.04 (−0.41; 0.35)

*Repeated measure correlation coefficient.

#Correlation between differences of measurements between two subsequent visits of a patient.

AChR-+, acetylcholine receptor antibody positive MG-patients, CI, confidence interval, MG-ADL, MG activity of daily life score, MuSK+, muscle specific positive MG patients, *n*, number of included patients, QMG, quantitative myasthenia gravis score.

Anti-LRP4-Abs positive patients had the lowest sNfl levels compared to the other MG subgroups. Adjusting for all other variables in this model, thymectomy and disease duration had no relevant effect on sNfl levels (Figure 3).

There was a trend for lowest sNfl levels in patients receiving symptomatic monotherapy with cholinesterase inhibitors (median of 7.00 pg/ml, IQR 5.8–21.0), followed by patients on corticosteroid monotherapy (median of 10.6 pg/ml, IQR 5.5–20.5), standard immunosuppressive therapy (azathioprine, mycophenolate mofetil, methotrexate; median of 13.4 pg/ml, IQR 7.99, 24.65), and patients receiving escalation therapy with rituximab (median of 21.9 pg/ml, IQR 6.85–37.2). Adjusting for all other variables in the log-linear model, there was no relevant effect of immunosuppression at baseline. The use of escalation therapies at baseline is associated with lower sNFl levels (coefficient (coefficient= −17.4; −52.1; 42.3), but due to uncertainty in this estimate, this finding cannot be generalized (Figure 3).

### 3.4. sNfl levels and disease severity

**Figure 4:**
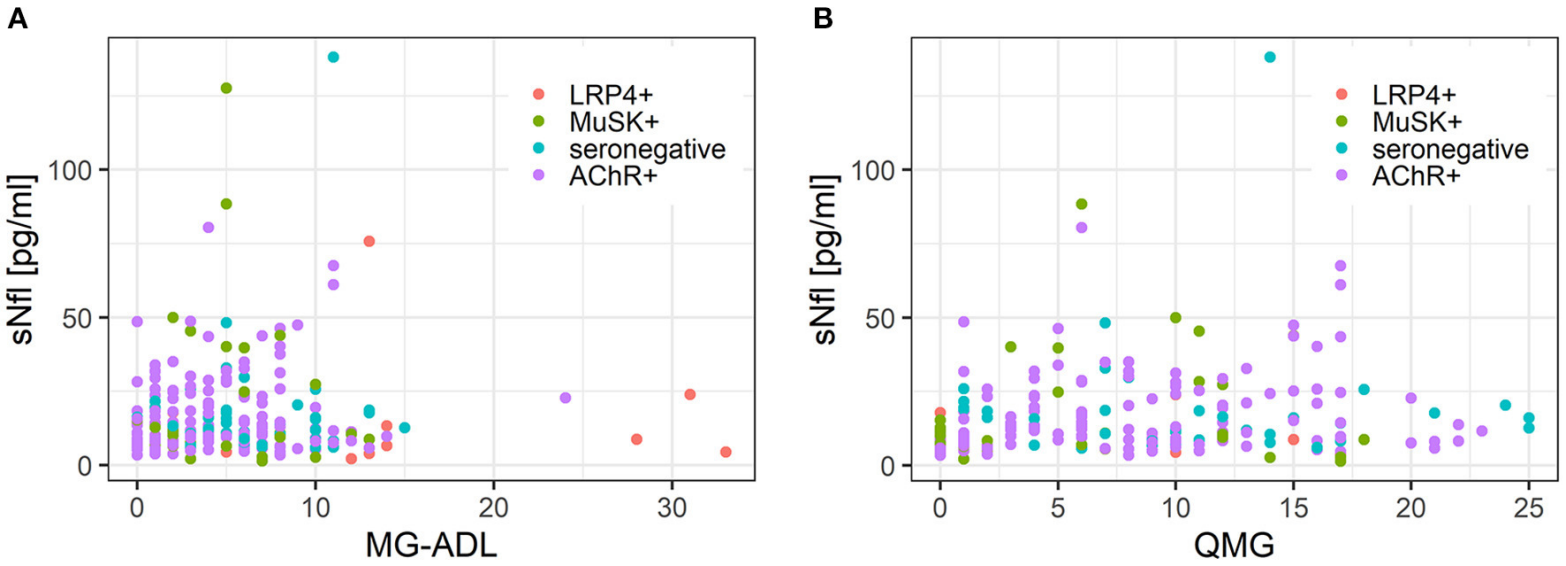
**(A)** Scatterplot of the association between sNfl levels and disease severity measured with QMG score in MG subgroups. **(B)** Scatterplot of the association between sNfl levels and disease severity measured with MG-ADL score in MG subgroups. AChR-+, acetylcholine receptor antibody positive MG-patients, LRP4+, lipoprotein related peptide 4 positive MG-patients, MuSK+, muscle specific positive MG patients, sNfl, serum neurofilament light chain, QMG, quantitative myasthenia gravis score, MG-ADL = myasthenia gravis activity of daily life score.

MG-ADL scores and sNfl levels did not correlate [values at baseline only; *r* = 0.03 (−0.14; 0.21)] (Figure 4A) or correlated weakly negatively [all available measurements; −0.11 (−0.30; 0.09)]. In contrast, there was a moderate positive correlation between QMG scores and sNfl levels at baseline [0.18 (0.00; 0.36)] (Figure 4B), whereas this was not found when all follow-up visits were taken into account (Table 2). When investigating the association between individual changes of sNfl with changes in MG-ADL and QMG scores, we found a negative correlation with both scores, but higher for MG-ADL [−0.20 (−0.48; 0.12), and QMG (−0.04 (−0.23; 0.16)] (Table 2). In a mixed log-linear regression model, sNFl levels were not relevantly associated with either MG-ADL or QMG levels (Figure 3).

## 4. Discussion

In this explorative cohort and case–control study, we demonstrated that sNfl levels of patients with MG were relevantly higher in comparison to controls. After adjusting for age and sex, median sNfl levels were highest for seronegative patients compared to controls. We did not find robust associations between sNfl levels and clinical disease severity measured as QMG or MG-ADL score. Therefore, our data suggest that sNfl is not a suitable biomarker for monitoring individual disease activity in MG.

There is limited evidence for neurogenic involvement in MG, but early histopathological studies suggested neurogenic changes in muscle biopsies (18), which was confirmed in a recent review regardless of MG subtype (17). One potential cause is complete or partial atrophy of muscle fibers based on permanent local acetylcholine (ACh) deficiency because ACh could exert a trophic influence on muscle fibers in addition to transmitting the impulse at the NMJ (17, 18). The pathophysiological mechanisms leading to ACh deficiency are well known: direct blockade of the receptors through MG auto-Abs, complement activation leading to destruction and loss of AChR content at the postsynaptic membrane, and depletion of AChR receptors by Abs-mediated crosslinking (29, 50, 51). These mechanisms depend on the specific IgG auto-Abs subclasses, Abs titer, and specific epitopes (29, 51). Another explanation for a possible neurogenic change might be the increasing evidence for presynaptic involvement in the pathogenesis of MG (29, 52). Presynaptic proteins such as synaptic vesicle glycoprotein 2A (SVP2) are enriched in patients with MG (29). Notably, in the presence of high anti-AChR-Abs titers, these presynaptic proteins may be activated as a bystander effect of complement activation (29). An example that complement activation can lead to bystander-induced neuronal injury was recently described in aquaporin-4-positive neuromyelitis optica suggesting that a complement-bystander injury may be a general mechanism for early neuronal injury (28). Nfl, a cytoskeletal protein of the presynaptic membrane, could thus be released into extracellular fluids and potentially serve as a biomarker for NMJ destruction.

We have found that levels of sNfl in the group of patients with MG were found to be on average 35% higher than those of controls. In the subgroup of patients with seronegative MG, they were even 44% higher than in controls. However, precise cutoff values to use sNFl as an additional marker for diagnosis need to be defined in analogy to other neuroinflammatory and neurodegenerative disorders. Since sNfl is a sensitive but unspecific marker of axonal loss, its potential diagnostic value lies mainly in distinguishing between a healthy and pathological state, as well as between diseases with different neurodegenerative damage potential. The best evidence for reliable cutoff values exists for ALS, where an sNfl value of 62 pg/ml was shown to have a sensitivity of 85.5% (95% CI 78–91.2%) and specificity of 81.8% (95% CI 74.9–87.4%) to discriminate ALS mimics such as chronic inflammatory demyelinating polyneuropathies (CIDP) or multifocal motor neuropathy (MMN) (53). In CIDP and MMN, a median sNfl value of about 28 pg/ml was described, although the majority of the patients were receiving immunosuppressive therapy at the time of sampling (13, 14, 54).

sNfl levels in blood depend on age, increasing by an average of 2.2% per year between the ages of 18 and 70 years in controls, mainly due to physiological age-dependent neuronal loss, which needs to be considered when defining cutoffs (55, 56). Therefore, the establishment of age-dependent thresholds for sNfl concentration is also necessary for its diagnostic use. As the difference in median age between patients with MG and controls was 6.5 years, we adjusted for age in our analyses to minimize this potential confounder. In addition, although the association between disease duration and sNfl is weak, the association with age is already taken into account, we adjusted for this variable as it is yet a potential confounder.

We found a tendency toward higher sNfl levels in anti-AChR-Abs positive patients as compared to the other subgroups. This finding might be related to the different pathogenetic mechanisms at NMJ, depending on the causative autoantibody. Anti-AChR-Abs predominantly belong to the IgG1 and IgG3 subtypes (22, 57). The binding of these antibodies results in the activation of the classical complement pathway with the assembly of the membrane attack complex (MAC) leading to local membrane damage and loss of AChRs at the NMJ (22). Especially in the presence of high anti-AChR-Abs titers, presynaptic proteins may be activated as a bystander effect of complement activation (29), leading to Nfl release in extracellular fluids, explaining the tendency of higher levels of sNfl in patients with positive anti-AChR-Abs status. The neuromuscular terminal seems to be enriched with proteins critical for NMJ structure and function, including cytoskeleton-associated proteins, like Nfl (24–27). In contrast, anti-MuSK-Abs belong mainly to the IgG4 subtype (58), which is not able to activate the complement system and act directly pathogenic by blocking the natural activation of MuSK, leading to progressive loss of AChRs from the motor endplate (22). This might explain the tendency of lower levels of sNfl in this subgroup. Additionally, muscle biopsies of patients with anti-MuSK-Abs MG show myopathic signs, whereas neurogenic features and atrophy are more frequently found in patients with anti-AChR-Abs-positive MG (17, 18, 59, 60). Additionally, we found a moderate positive correlation between sNfl levels and levels of anti-AChR-Abs and anti-MuSK-Abs titers, which is an interesting clinical finding as data on the correlation of clinical severity with Abs-titer remains controversial (61–63).

It should be emphasized that after adjustment for age and sex, we found the highest median sNfl levels in seronegative MG patients. This observation might support the recent finding that in up to 60% of previously negative-tested patients with MG clustered auto-Abs against the AChR at the NMJ can be detected with cell-based essays (64–66), and that complement deposition is found at the NMJ (67). There is *in vitro* evidence that clustered auto-Abs can strongly activate complement-causing severe NMJ destruction and muscle weakness in a passive transfer MG rat model (29), which might explain why seronegative MG patients presented with the highest sNfl levels in our study. It might also provide novel opportunities for biomarkers of critical exacerbation in this understudied patient population (68). Nevertheless, the accessibility of cell-based essays is still limited and confined to specialized research centers. Thus, sNfl might be of diagnostic value, since a seronegative Abs-status carries is the risk of diagnostic uncertainty, but it is also of therapeutic relevance given an increasing antibody-specific treatment (3, 4).

It is established that MG is most active in the first 2–3 years after diagnosis (51). These findings relate to previous research on sNfl in ALS and multiple sclerosis, where sNfl levels are higher in active disease stages serving as an individual marker of disease progression (40, 69). However, although we found a positive association of sNfl with QMG scores at baseline, in our longitudinal analyses, we found contradictory results regarding the associations of sNfl levels with the MG-ADL and QMG scores in our study population. Therefore, no clear relationship of sNfl with parameters of disease activity can be drawn based on our results. Nevertheless, it should be mentioned that the majority of our study population received immunosuppressive therapies at sampling time and that the main proportion of patients experienced no significant change in QMG and MG-ADL scores during follow-up, which might have had a confounding effect.

We did not find a definite association between sNfl levels and MG-specific treatment. No association was shown for thymectomy history. Patients without immunosuppressive therapy tended to have lower nNfl levels while patients with escalation therapy had the highest sNfl levels. However, we cannot reliably assess the effects of MG-specific treatments on sNfl levels because of the small number of patients in the treatment subgroups. This should be investigated in larger, prospective studies, preferably in a treatment-naïve cohort.

There are several limitations to our study. Our cohort study was rather small with respect to the antibody subgroups, although we included a rather high number of patients with MG in our study. In this exploratory study, we nevertheless analyzed samples from patients with different Abs status, thymus pathology, and age at onset. In addition, the majority of included patients were not treatment naïve and therefore heterogeneous with respect to treatment regime and disease duration. Although patients with MG with pre-existing neuro-inflammatory and neurodegenerative diseases, as well as recent ICU stay, were excluded from the study, we cannot exclude the possibility that some patients, as well as controls, may have suffered from a subclinical neurodegenerative or neuroinflammatory disease that may have influenced sNfl levels. The control population was small and the median age was lower than in the MG population, for which we, however, adjusted in all analyses. In addition, age-adjusted z-scores were derived for cases and controls based on a reference database (40–42) to correct for age.

In conclusion, this exploratory cohort and case–control study demonstrates that sNfl levels were relevantly higher in patients with MG compared to controls. sNfl levels were descriptively higher in anti-AChR-Abs positive patients than in other MG subgroups. Adjusting for age and sex, seronegative patients had the highest sNfl levels in comparison to controls. Although sNfl levels are significantly increased in patients with MG compared to controls, the difference is small. Furthermore, sNfl levels do not correlate robustly with the clinical severity of MG. Therefore, sNfl is not a suitable biomarker for monitoring individual disease activity in patients with MG. However, as we found interesting differences in sNfl levels associated with auto-abs status, our study may prompt further studies in larger, treatment-naïve cohorts to evaluate the potential of sNfl as a biomarker of disease progression in specific MG subgroups.

## Data availability statement

The raw data supporting the conclusions of this article will be made available by the authors, without undue reservation.

## Ethics statement

The studies involving human participants were reviewed and approved by Ethics Committee of the Charité—Universitätsmedizin Berlin (EA1/281/10). The patients/participants provided their written informed consent to participate in this study.

## Author contributions

FS: designed and conceptualized the study, had a major role in the acquisition of data, analyzed and interpreted the data, drafted the manuscript for intellectual content, and takes full responsibility for the integrity of the data analyzed. AA and BH: analyzed and interpreted the data and revised the manuscript for intellectual content. PM: acquisition of data, interpreted the data, and revised the manuscript for intellectual content. SH and SL: acquisition of data and revised the manuscript for intellectual content. CM: laboratory analysis and revised the manuscript for intellectual content. AM: designed and conceptualized the study, acquisition of data, interpreted the data, and revised the manuscript for intellectual content. All authors contributed to the article and approved the submitted version.
