# Supplementary material for: Serum neurofilament light chain in myasthenia gravis subgroups: An exploratory cohort and case–Control study
Source: Front Neurol. 2023 Jan 11;13:1056322. doi: 10.3389/fneur.2022.1056322 (PMC9875128; doi:10.3389/fneur.2022.1056322)
Supplement: Supplementary file 1 [file Table_1.docx]

**Supplementary Table 1: Baseline characteristics and medical history of myasthenia gravis patients** **with sNFL levels > 95 % quantile**

|  | MG patients with snfl >95% quantile | Total MG cohort |  |
| --- | --- | --- | --- |
| n (%) | 7 | 134 |  |
| Sex  Female, n (%) | 5 (71%) | 85 (63%) |  |
| Age at Time point of Sampling, Median (IQR)  ANTIBODY STATUS  ANTI-AChR-ABS  ANTI-MUSK-ABS  ANTI-lrp4-abs  seronegative | 54 (45.5-67.5)  3 (44%)  2 (28%)  2 (28%)  0 (0%9 | 52.5 (39-68)  79 (59%)  18 (13%)  11 (8%)  26 (19%) |  |
| Disease duration (years), MEdian (IQR) | 2.0 (2.0-10.5) | 4.0 (2.0-10.5) |  |
| History of myasthenic exacerbation/CRISIS, n (%)  (missing) | 1 (20%)  (2) | 32 (25%)  (11) |  |
| MGFA classification at time point of sampling, Median (IQR)  (missing) | 3.0 (1.0-3.0)  (1) | 2 (1.0-2.0)  (1) |  |
| QMG, median (IQR)  (MISSING) | 6 (5.0-15.0)  (2) | 7 (2.75-13.0)  (10) |  |
| MG-ADL-Score, Median (IQR)  (MIssing) | 8 (5.75-10.25)  (1) | 5 (2.0-8.0)  (3) |  |
| History of thymectomy  Thymoma, N (%)  (missing) | 2 (29%)  0 (0%)  (0) | 52 (39%)  1 (2%)  (76) |  |
| MG-specific treatment at baseline , N(%)  Azathioprine  MycophenolatE mofetil  Methotrexate  Rituximab  Eculizumab  sNfl (PG/ML), MEDIAN (IQR) | 1 (14%)  2 (29%)  1 (14%)  1 (14%)  0 (0%)  49.8 (47.5-71.6) | 45 (34%)  15 (11%)  6 (4%)  4 (3%)  1 (1%)  11.2 (6.8-22.3) |  |

Results presented as median (IQR) or n (%). Disease duration is the time from diagnosis until baseline. *Abbreviations*: AChR-+=acetylcholine receptor antibody positive MG-patients, IQR = interquartile range; LRP4+ = lipoprotein related peptide 4 positive MG-patients, MG = myasthenia gravis, MG-ADL = MG activity of daily life score, MGFA = Myasthenia gravis foundation of America classification; MuSK+ = muscle specific positive MG patients, sNfl = serum neurofilament light chain, QMG = quantitative myasthenia gravis score; - = not applicable.
